# Influence of Quinine and Malaria over Pregnancy

**Published:** 1860-03

**Authors:** 


*Influence of Quinine and Malaria over Pregnancy.*

Dr. Davidson, of Arkansas, reports a case of threatened abortion, caused by malaria, and successfully treated with quinine. Dr. Davidson says: "Knowing the patient to have been the subject of intermittent fever the summer and fall previous, it occurred to me that perhaps malaria was the cause of the uterine disorder. I therefore administered between fifteen and twenty grains of quinine the next day. She had no return of pains for about three weeks, at the end of which time they came on again. I stopped them with laudanum, but they returned daily, until I gave quinine." * * * "I several times withheld the quinine for a day or two, when the pains would invariably return; but when it was administered regularly it never failed to keep them in check."

